# The Anti-cancer Effect of *Olea europaea* L. Products: a Review

**DOI:** 10.1007/s13668-021-00350-8

**Published:** 2021-03-08

**Authors:** Chrystalla Antoniou, Jonathon Hull

**Affiliations:** Faculty of Health and Life Sciences, University of the West of England, Coldharbour Lane, Bristol, BS16 1QY UK

**Keywords:** Olive, *Olea europaea*, Chemotherapeutic, Cancer

## Abstract

**Purpose of Review:**

The olive tree (*Olea europaea* L.) has featured as a significant part of medicinal history, used to treat a variety of ailments within folk medicine. The Mediterranean diet, which is rich in olive products, is testament to *Olea europaeas* positive effects on health, associated with reduced incidences of cancer and cardiovascular disease. This review aims to summarise the current literature regarding the therapeutic potential of *Olea europaea* products in cancer, detailing the possible compounds responsible for its chemotherapeutic effects.

**Recent Findings:**

Much of the existing research has focused on the use of cell culture models of disease, demonstrating *Olea europaea* extracts, and specific compounds within these extracts, have efficacy in a range of in vitro and in vivo cancer models. The source of *Olea europaeas* cytotoxicity is yet to be fully defined; however, compounds such as oleuropein and verbascoside have independent cytotoxic effects on animal models of cancer.

**Summary:**

Initial results from animal models are promising but need to be translated to a clinical setting. Treatments utilising these compounds are likely to be well tolerated and represent a promising direction for future research.

## Introduction

The olive tree (*Olea europaea* L.) and its products have been an important commodity throughout human history. Today, 98% of olive products are cultivated in the Mediterranean basin and are an important part of the economy of the region [1]. The value of the olive tree, however, extends past economics to its nutritional and medicinal properties [2]. Olive tree products were used in traditional medicines to treat a variety of ailments such as fever [3, 4]. In more recent times however, it has demonstrated possibilities in cancer prevention. Cancer rates in the Mediterranean are notably lower than other western countries, with cancer rates in Greece of 279.8 per 100,000 compared to 352.2 and 319.2 in the USA and UK respectively. It is expected that this protection is a result of dietary differences between these populations, as the Mediterranean diet is consistently associated with reduced incidences of cancer and cardiovascular disease [5, 6].

Academic studies of the beneficial properties of olives initiated in 1854 with the work of Daniel Hanbury, who noted that a decoction of olive leaf was effective in reducing fevers associated with malaria [4, 7]. Many pharmacological reports have since exhibited the potential of olive-based extracts to relieve arrhythmia, increase blood flow, lower blood pressure, inhibit viral activity and prevent intestinal muscle spasms [8, 9]. Scientific studies looking at the anti-proliferative components of certain flora, have recently demonstrated this potential within *Olea europaea* products, largely from in vitro studies using extracts in addition to testing isolated compounds from *Olea europaea* products [7]. This review will summarise the key studies that relate to *Olea europaea* extract’s anti-cancer effects and discuss compounds chosen for their high abundance within *Olea europaea*, revealing their anti-proliferation properties.

## *Olea europaea* Extracts

The preponderance of research in this field has focussed on the use of cell culture models of disease, with efficacy in a range of these cancer models. Fares et al*.* [10] studied the effect of olive leaf extract (OLE) on human lymphoblastic leukaemia cell line, Jurkat. The results reported a 78% inhibition of Jurkat cell proliferation at a concentration of 4 μg/mL at 48 hours. However, the study could not elucidate the mechanism of apoptosis. OLE has demonstrated efficacy against the myelogenous leukaemic cell line, K562. After a 72-h treatment with 150 μg/mL of OLE, the cell proliferation was inhibited to 17%, whilst also demonstrating a significant decrease in viability [11].

Examination of OLE on a pancreatic cancer cell line (MiaPACa-2) demonstrated efficacy in culture [12]. At concentrations of 200 μg/mL, the OLE was able to reduce cell viability to less than 1% compared with controls. Coccia et al*.* [13] observed the cytotoxic effect of extra virgin olive oil extract on two different bladder cancer cell lines. It was demonstrated that oil significantly decreased proliferation in T24 and 5637 bladder cell lines in a dose dependent manner. Cell viability of T24 cells was decreased by up to 90% with 100 μg/mL oil, with an IC_50_ of ~32 μg/mL, with similar treatment efficacy in 5637 cells [13]. Furthermore, the cell cycle progression was monitored by flow cytometry, that observed a growth arrest at the G2/M phase after treatment in both cell lines. In the T24 cell line, a decrease was observed in the G0/G1 phase and an increase in the sub-G1 fraction, indicative of induction of apoptosis. Western blot analysis of pro-caspase-3, -9 and PARP-1 further demonstrated an apoptotic effect of oil [13]. Olive oil production generates waste materials often referred to as olive mill wastewater. This wastewater still contains many of the same compounds present in olives. Recently, Baci et al*.* [14•] tested this wastewater on PC-3 prostate cancer cells in culture. The results demonstrated wastewater was able to inhibit cell proliferation, adhesion, migration and invasion. At a molecular level, the wastewater was observed to inhibit NF-κB signalling in addition to reducing pro-angiogenic growth factors, VEGF, CXCL-8 and CXCL-12 production. The results demonstrated that even from the lowest dilution tested (1:5000), wastewater was able to inhibit PC-3 cell proliferation [14•].

Isleem et al*.* [15] demonstrated the importance of extraction techniques on OLE efficacy. In this study, OLE was mixed with either deionised water or 80% methanol, and these extracts were subsequently tested individually in a dose- and time-dependent manner on the human breast cancer cell line MCF-7. After a 48-h treatment, the deionised water extract had an IC_50_ value of 182 μg/mL whilst the methanol extract was 135 μg/mL, versus relevant controls. Furthermore, the source of the products are often not comparable, something which is supported by in vivo studies. For example, a study by Escrich, Moral and Solana [16] investigated how diets rich in extra virgin olive oil reduce the risk of breast cancer compared with diets high in corn oil. Olive oil induced molecular changes in tumours that resulted in higher rates of apoptosis, lower proliferation and lower DNA damage. Furthermore, Milanizadeh and Reza Bigdeli [17] demonstrated a reduction in mammary cancer weight and volume after a treatment of 150 and 225 mg/kg/day of OLE in a breast cancer mouse model. This reduced growth was proposed to be related to the polyphenol content of OLE, resulting in an increase of antioxidant enzyme activity including superoxide dismutase and catalase. The mechanisms responsible for inhibited tumour growth are likely to stem from OLE polyphenols blocking the cell cycle in G1/S through reducing COX2 and cyclin D1 expression, which results in the reduction of cell growth and proliferation [17, 18].

Whilst the source of cytotoxicity of OLE has yet to be fully defined, oleuropein is the most abundant compound in olive leaves, followed by hydroxytyrosol, luteolin, apigenin and verbascoside. Oleuropein is a heterosidic ester of elenolic acid and dihydroxyphenylethanol. Hydroxytyrosol (3,4-Dihydroxyphenyl ethanol), is the principal degradation product of oleuropein [19]. Within unprocessed olive fruit and leaves, oleuropein is more abundant, whereas hydroxytyrosol is present in higher amounts of processed olive fruit and leaves [20]. This change in concentration takes place due to chemical and enzymatic reaction that occurs during maturation of olive products or as a result of processing. One key component of olives and olive oil is their fatty acid composition. Olive oil largely consists of triacylglycerols (98-99%), a group of glycerol esters with varying fatty acids [21]. The main fatty acid in olive oil is oleic acid; however, it also contains linoleic acid, palmitic acid, palmitoleic acid, stearic acid and the triterpene maslinic acid. A variety of amphiphilic and lipophilic microconstituents exist in olive oil including, tocopherols, squalene, phytosterols, and phenolic compounds [22]. These compounds are summarised in Table 1.

**Table 1:**
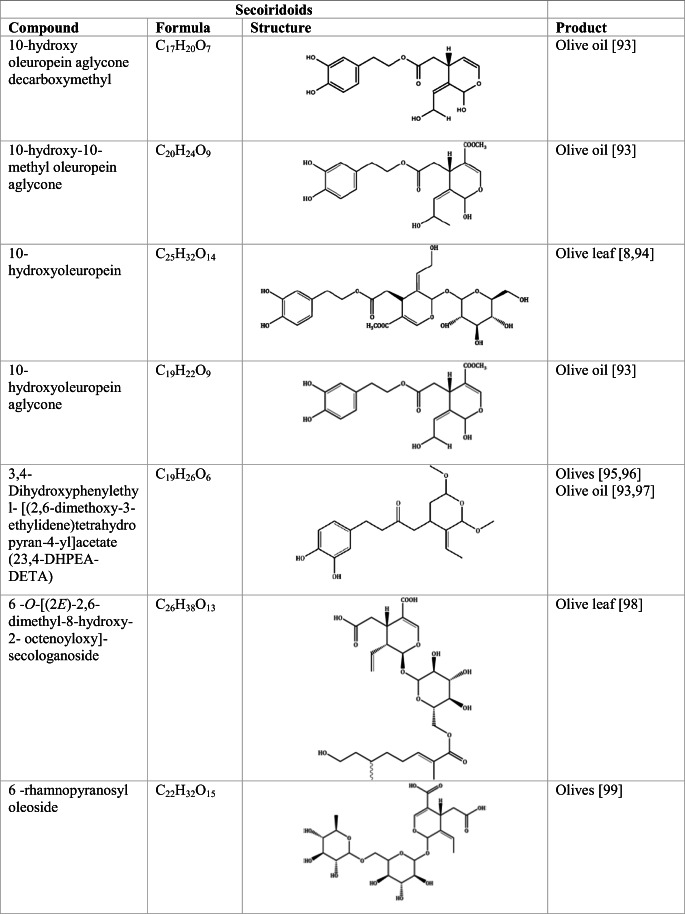

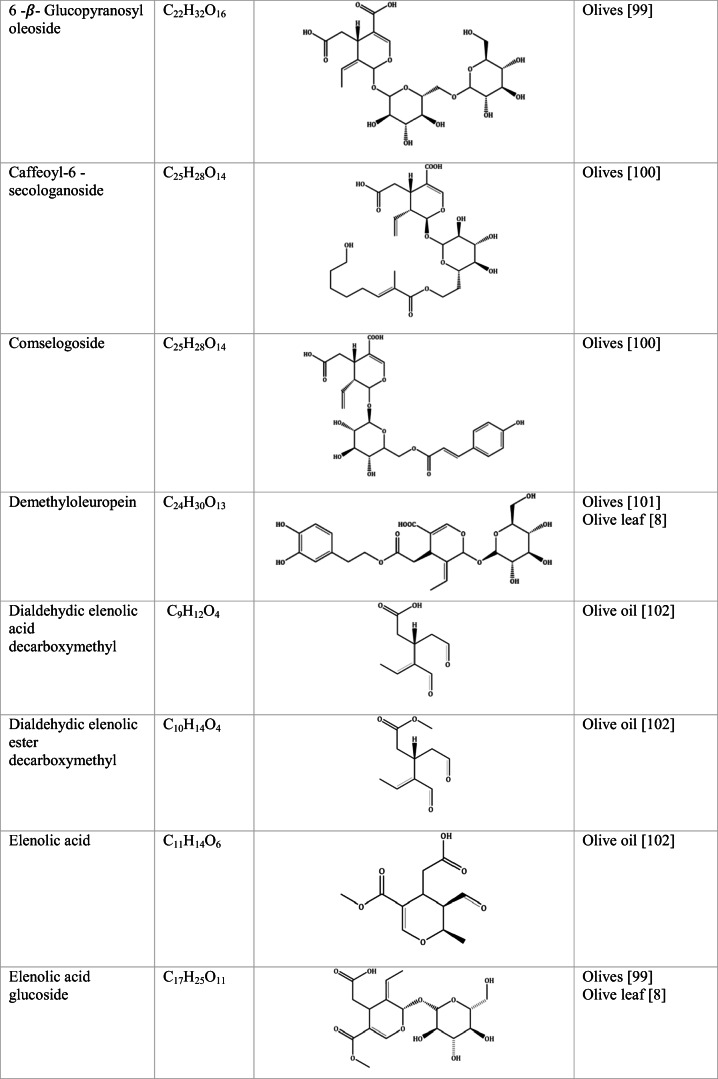

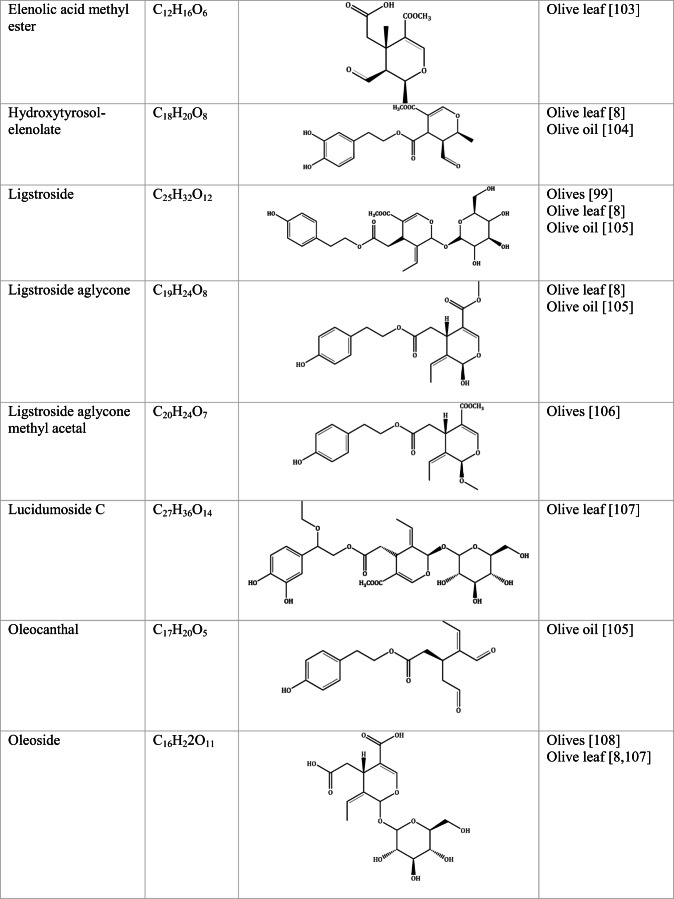

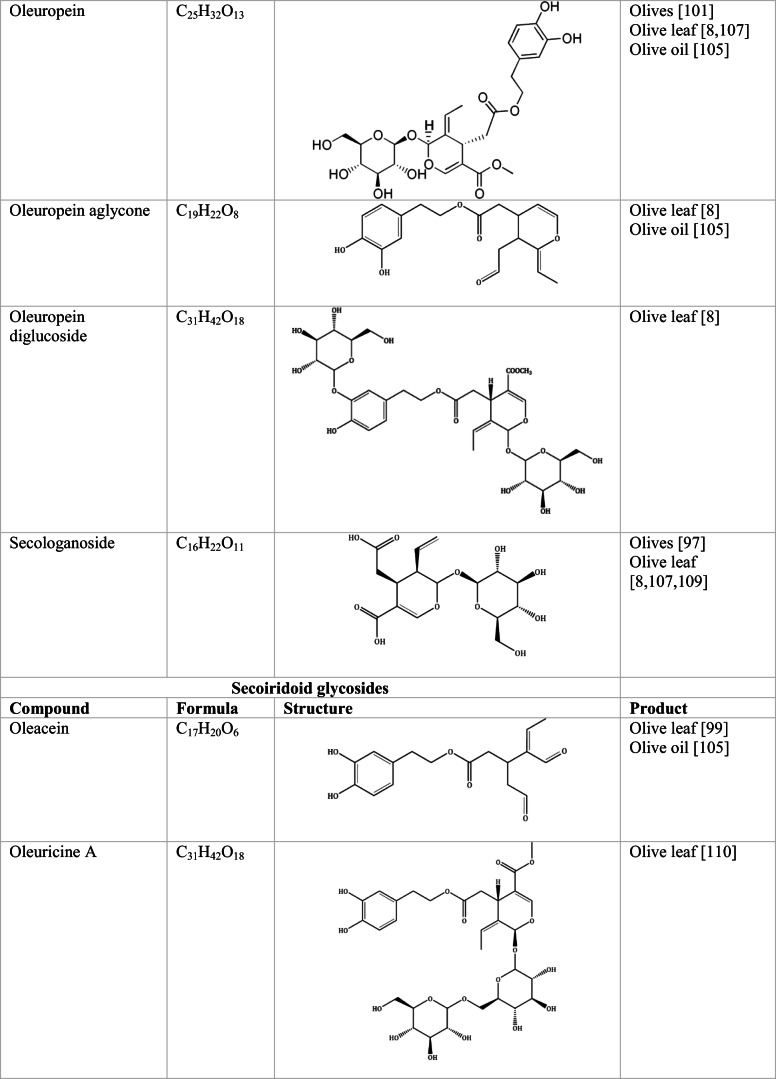

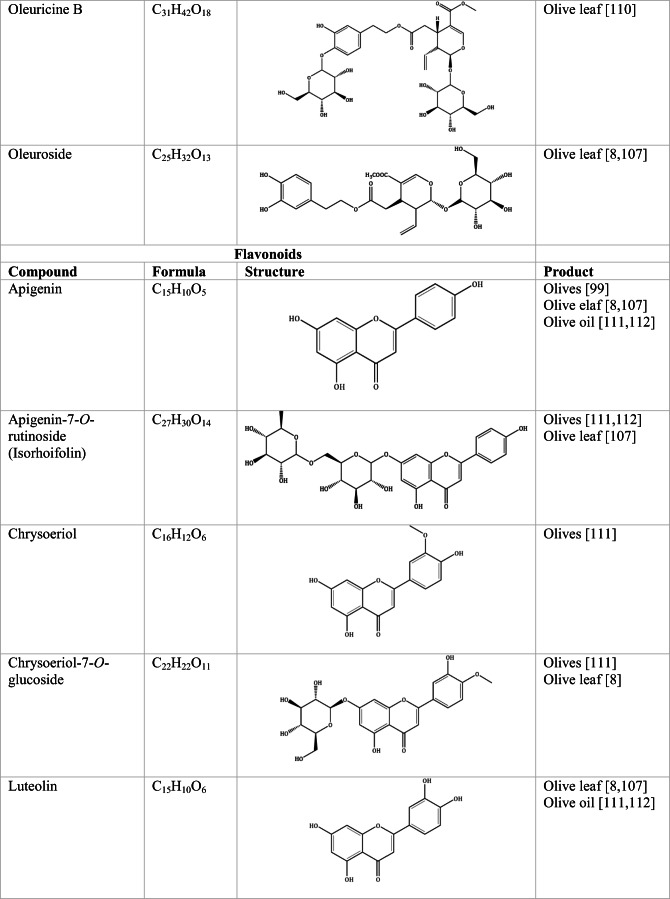

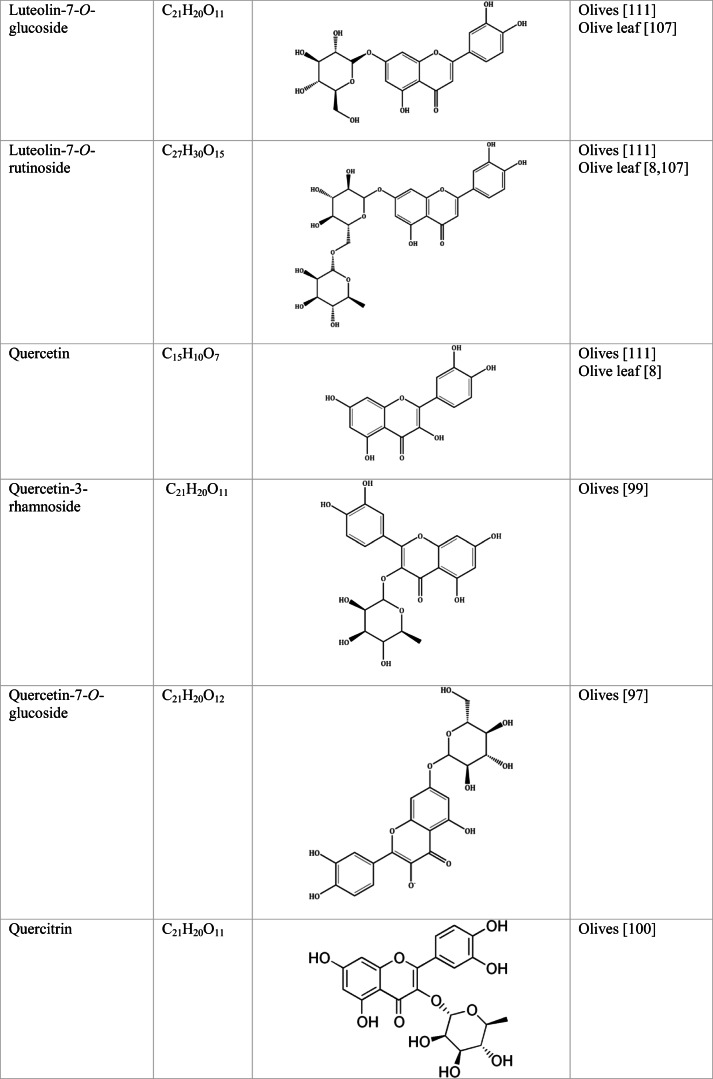

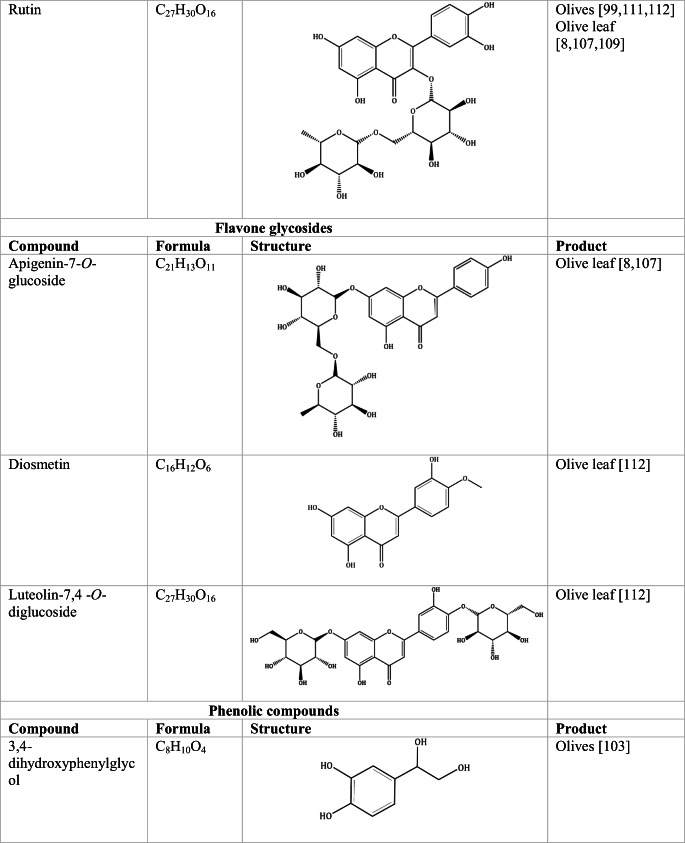

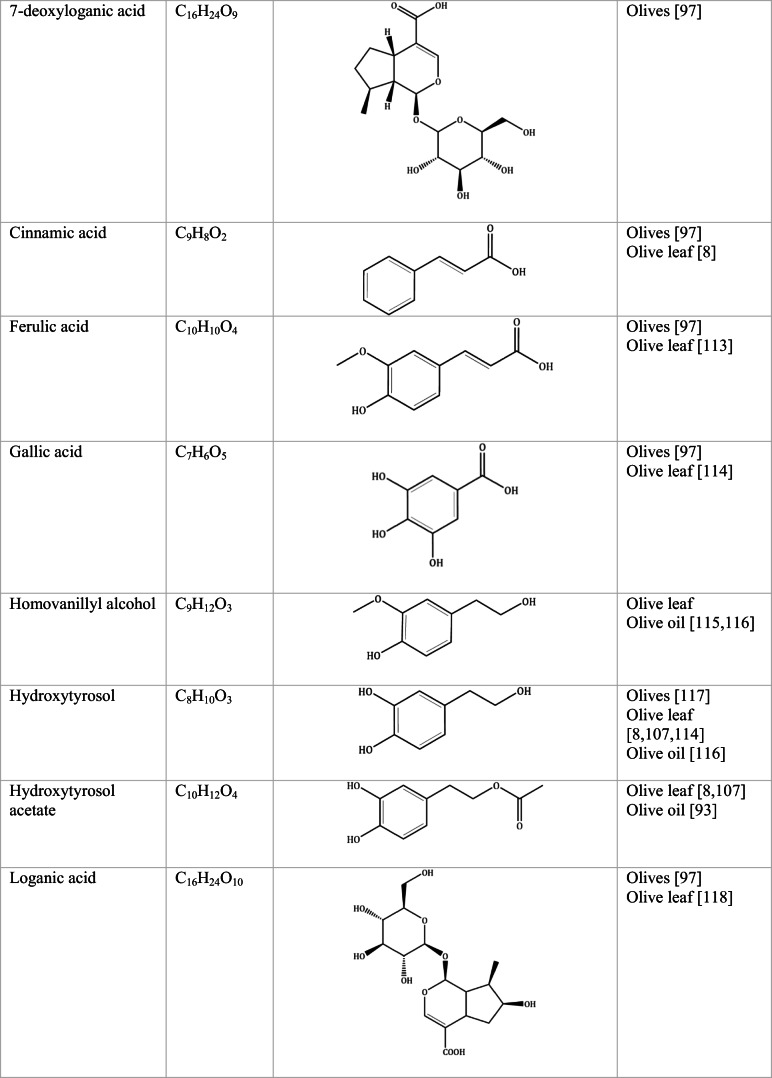

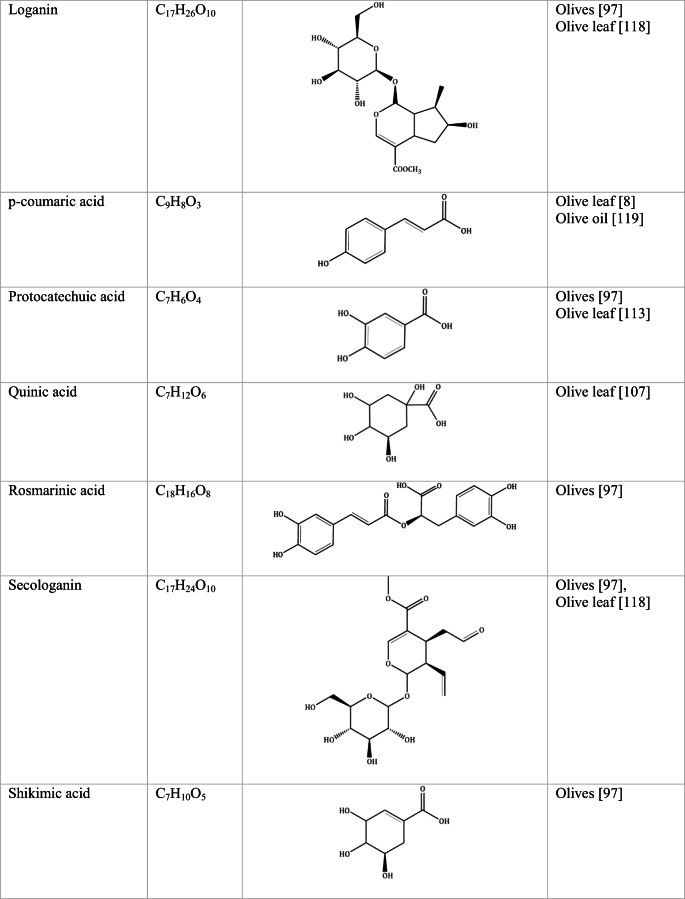

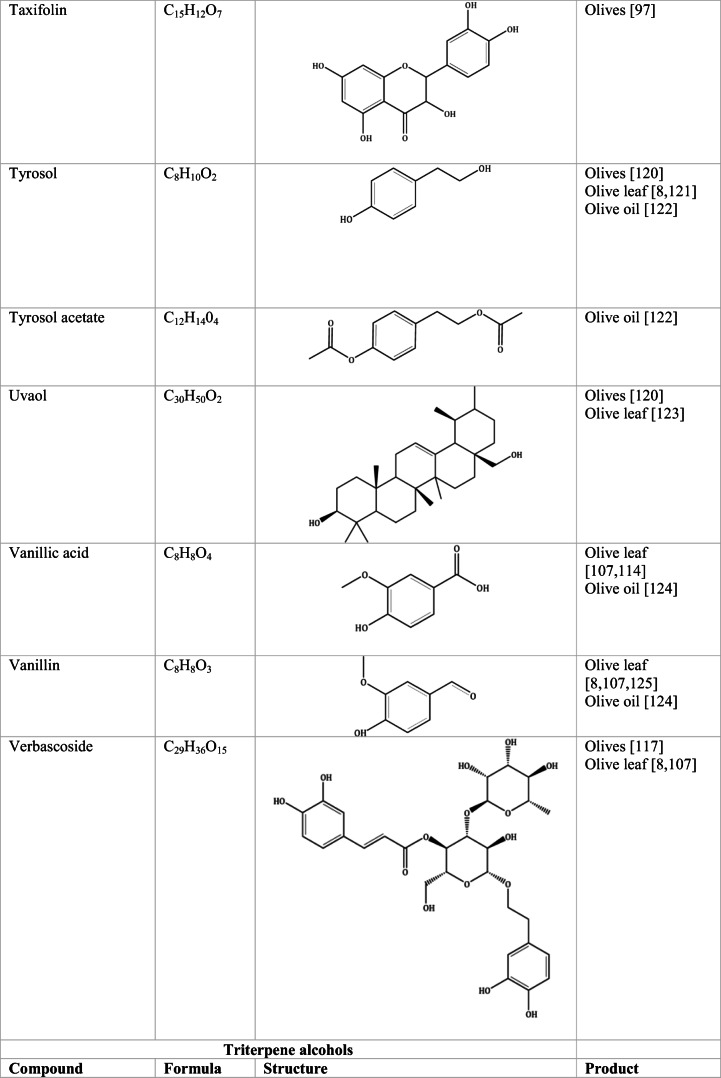

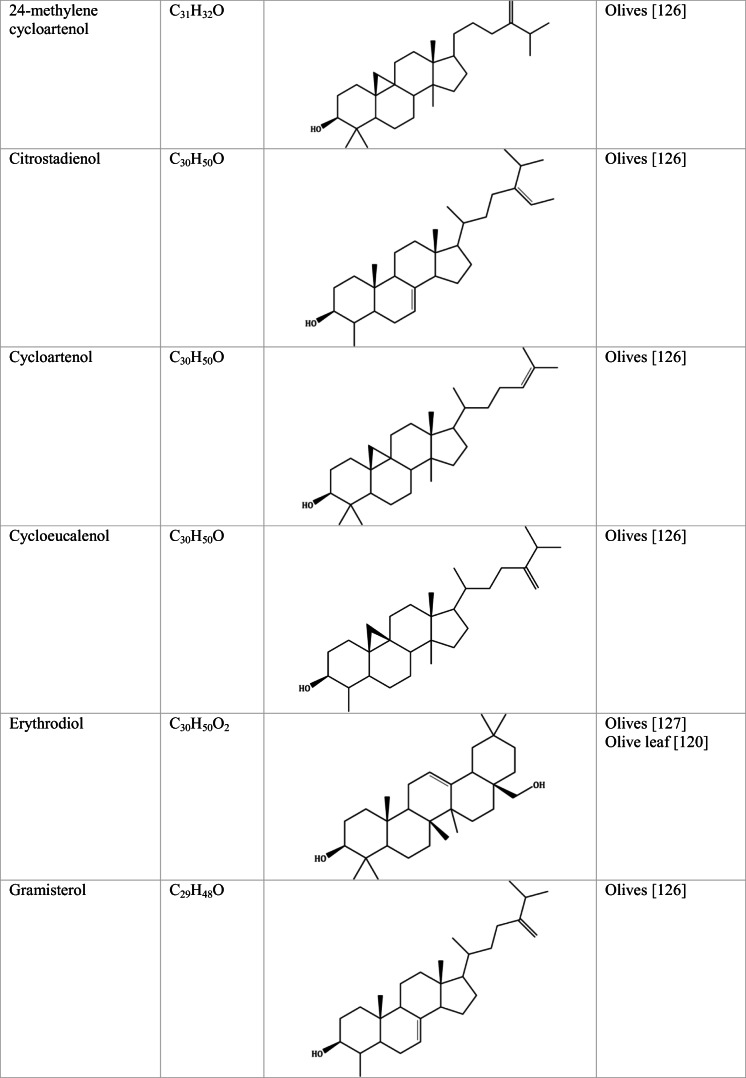

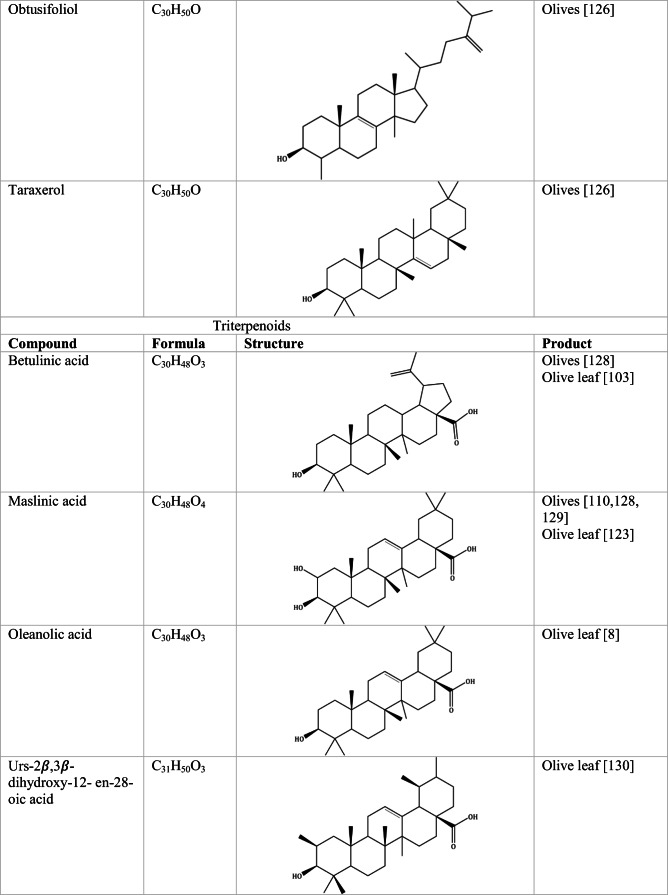

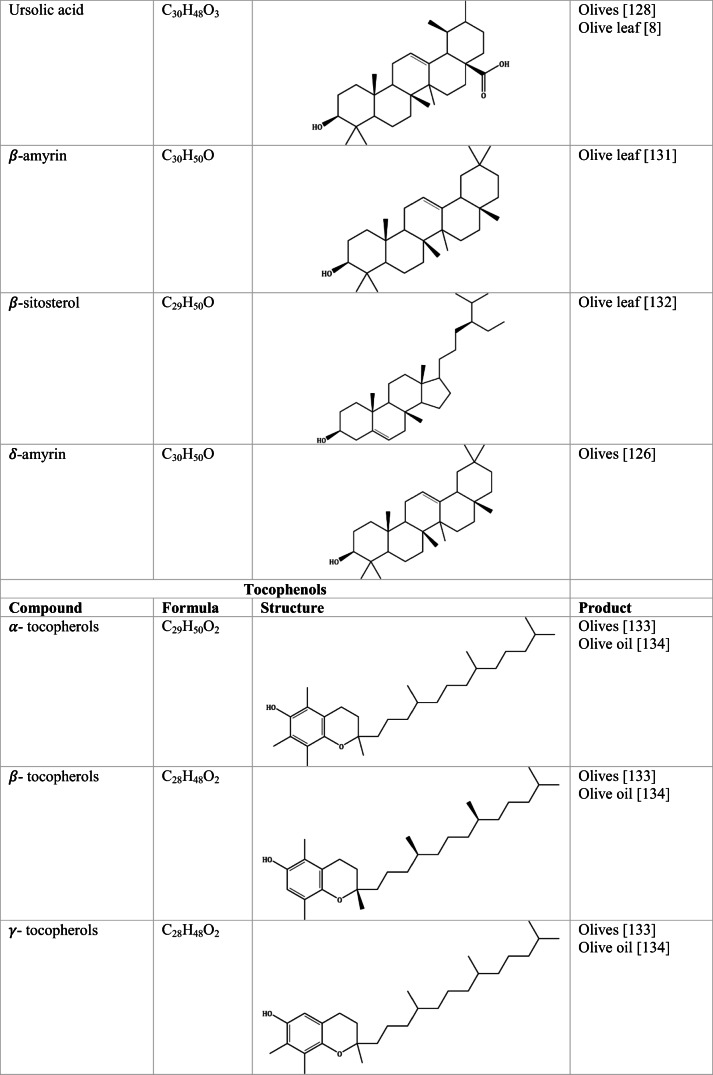

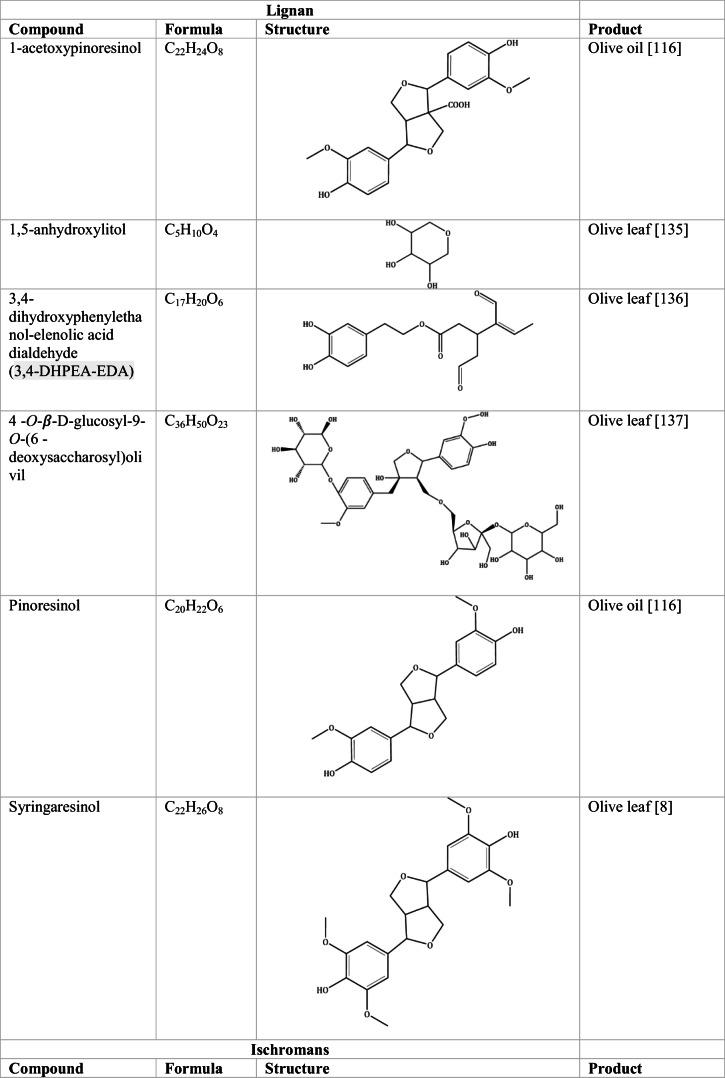

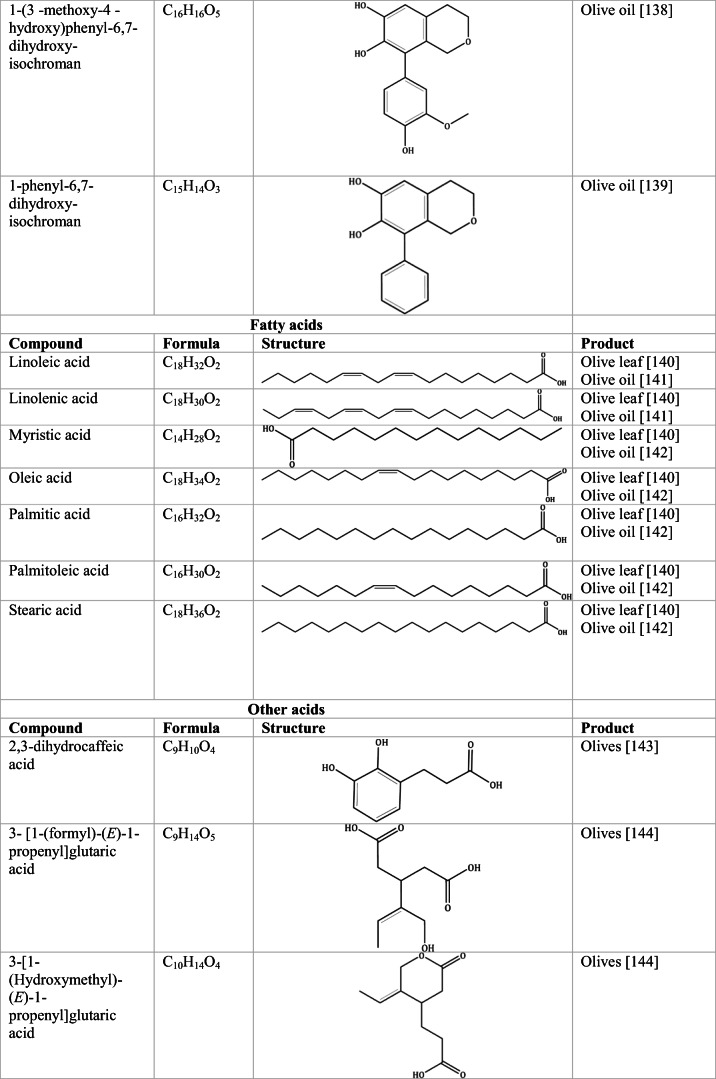

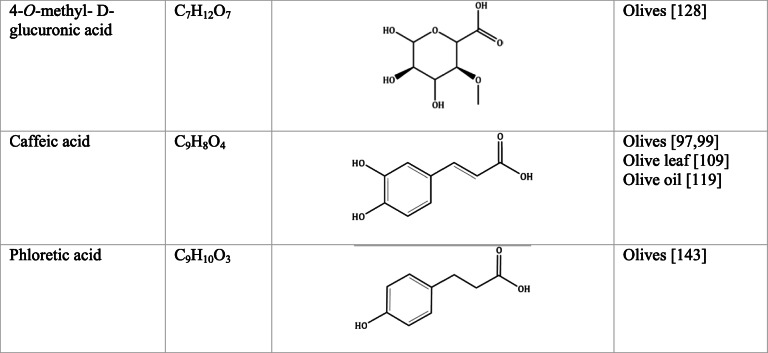
Phytochemicals isolated from Olea europaea (Olive tree) products

## Polyphenols

Polyphenols are a family of micronutrients, named due to the presence of multiple hydroxylated phenol rings in their structure. These compounds are broadly water soluble and include plant pigments and tannins [23]. The proposed mechanisms of action associated with polyphenols are largely related to their antioxidant activity, directly leading to a reduction in reactive oxygen species (ROS) [24, 25]. Phenols, oleuroepsides and flavonoids have proven to demonstrate significant antioxidant activity towards free radicals, because of the redox properties of their phenolic hydroxyl groups and the structural relationships within their chemical structure [9]. Other research has demonstrated the ability of polyphenols to modulate the human immune system, resulting in increased regulatory T cell and splenocyte production, in addition to reducing oxidative burst activity of neutrophils [26].

The concentration of polyphenols in olive products can depend on factors including climate, cultivation, maturity, rootstock, agricultural practices and the method of extraction, separation and quantification [27–29]. It was observed by Wang and Fordham [30] that the phenolic content of olives is season dependent, with olives harvested in the autumn having a higher polyphenol and carotenoid content, and therefore, a higher antioxidant capacity. Pereira [31] highlighted that black olives present a higher antioxidant capacity than their green counterpart, due to higher concentrations of phenolic compounds in black olives. Thus, the different variety of olives and processing result in polyphenol content varying significantly in different olives and consequently olive oil. Research studying the olive leaves has observed notable variety and quantity of polyphenols compared with olive oil, notably oleuropein and hydroxytyrosol [32]. Just as with olives, the chemical composition of olive leaves changes under conditions such as climate, country of origin, moisture, storage condition and soil content [33].

The reduced cancer rates associated with the Mediterranean diet and the history of *Olea europaea* products in traditional medicine have made olive tree products the focus of phytochemical research [34–36]. Studies have identified some compounds from olive tree products for a deeper investigation of their cytotoxic effect. The most ubiquitous compounds are the phenolic compounds, flavonoids, secoiridoids and secoiridoid glycosides. However, compounds such as flavanones, flavone glycosides, triterpenes, benzoic acid derivatives, biophenols, sterols, sugars, xylitol and isochromans have clear biological activities [28].

## Secoiridoids

Secoiridoids are uniquely present in plants of Olearaceae family [37]. Secoiridoids are monoterpenoids based on the 7,8-secocyclopenta[c]-pyranoid skeleton. In plants, it is possible that they derive from iridoids that are cleaved with redox enzymes and subsequently undergo several secondary modifications (oxidation, epoxidation, esterification) of the generated hydroxyl groups within the main skeleton. This produces a group of compounds, which constitute the class [38].

### Oleuropein

Oleuropein is recognised to have antiviral, antimicrobial and antifungal properties [39]. Moreover, oleuropein has noted hypoglycaemic and hypotensive properties, and is a powerful antioxidant [40, 41]. Oleuropein has proven to exert protective properties against cancer and heart disease, in addition to supporting immunoregulatory actions [42]. Oleuropein inhibits migration and proliferation of human tumour cells in culture. Hamadi and Castellon [43] demonstrated that oleuropein irreversibly rounds cancer cells, preventing motility, replication and invasiveness, whilst these effects were reversible upon treatment removal in normal cells. The study [43] demonstrated this reversibility through carrying out additional washing tests on Matrigel-free cultures of RPMI-7951 melanoma cells in addition to normal cells. After 48 h of extensive washing, normal cells flattened out and again became mobile, whilst cancer cells remained immobilized. This work was supported in vivo, when 1% oleuropein was administered to mice orally within drinking water leading to tumour regression in 9-12 days [43]. The cell rounding triggered by oleuropein is linked to the disruption of the actin cytoskeleton and actin filaments. In Hamadi and Castellon’s [43] study, it was established that this effect was somewhat offset by the addition of glucose in the culture media. Due to oleuropeins glucose moiety, it is likely that oleuropein enters the cell through glucose transporters (GLUTs). Removing oleuropeins glucose moiety with β-glucosidase decreases its anti-proliferative activity, demonstrating that entry of oleuropein into the cell is at least partially dependent on the glucose moiety [43]. Human malignancies are associated with elevated glucose uptake and enhanced expression of several GLUT isoforms [44]. This explains how normal cells can reverse the rounding effects of oleuropein treatment, as normal cells have low expression of GLUTs [45, 46].

Numerous studies have demonstrated oleuropeins efficacy against certain breast cancer cell lines. Sirianni et al*.* [47] demonstrated the inhibition of estradiol-dependent activation of extracellular regulated kinase 1/2 (ERK 1/2) by oleuropein in the MCF-7 cell line. This is significant as estradiol stimulates the growth of several breast tumours, through induced cellular proliferation. It is established that oleuropein can reduce cell proliferation of MCF-7 and T47D cells, this reduce proliferation is associated with the activation of autophagy and suppression of migration and invasion; this is achieved through p62 downregulation, in addition to Beclin-1 and LC3II/LC3I upregulation [48]. Oleuropein increases the level of ROS and induces apoptosis via modulating NF-κB activation cascade, within MDA-MB-231 and MCF-7 cell lines [49]. Elamin et al*.* [50], treated breast cancer xenografts (in nude mice) *in vivo,* with a combination of doxorubicin and oleuropein. This treatment combination resulted in a downregulation of NF-κB, Bcl-2 and survivin, triggering apoptosis. All treatments significantly reduced tumour volume compared to untreated controls (tumour volume, 173mm^3^). However, combination therapy of doxorubicin and oleuropein (48.7mm^3^) had greater efficacy than either doxorubicin (69mm^3^) or oleuropein (79mm^3^) alone. Oleuropein has further demonstrated efficacy in numerous cancer cell lines such as colorectal, thyroid and lung [51–54]. Future work will be needed to apply this research to the clinical environment.

### Oleocanthal

Studies have outlined the potential of oleocanthal for cancer prevention in many cancer types. Akl et al*.* [55] demonstrated that oleocanthal can inhibit the growth of human breast cancer cell lines MCF-7, MDA-MB-231 and BT-474, whilst not affecting normal human cell growth of MCF10A. Possible mechanisms of action in these cell lines point to the blocking of cell migration, invasion and G1/S cell cycle progression. This mechanism occurs through the inhibition of Hepatocyte growth factor (HGF)-induced c-Met activation [55]. Oleocanthal was investigated for its antiproliferation activity in human melanoma cell lines, 501Mel and A375. It was determined that oleocanthal inhibits cyclooxygenase enzymes to exert important anti-inflammatory activities [56]. This study demonstrated that oleocanthal did not produce significant changes in human dermal fibroblast viability, signifying selective activity for cancer cells over normal cells. This selectivity has been associated with oleocanthals ability to induce lysosomal membrane permeabilization leading to apoptosis and/or necrosis. Cancer cells largely have weak lysosomal membranes compared to noncancerous cells, thus making them susceptible to cell death via lysosomotropic agents [57]. Fogli et al*.* [56] demonstrated oleocanthal induced cell growth inhibition in 501Mel and A375 cells in a concentration-dependent manner, with an IC_50_ of 13.6 and 20 μM respectively. It was demonstrated that oleocanthal downregulates Bcl-2, Erk1/2 and AKT signal transduction pathways [56, 58]. These pathways play a key role in oleocanthal-induced cytotoxicity in multiple myeloma cells. Moreover, the activation of the AKT pathway is closely associated with resistance to BRAF inhibitors in melanoma patients [59].

## Flavonoids

Flavonoids are secondary metabolites corresponding to polyphenols that have a diverse structure, taking the form glycosides or aglycones in fruits and vegetables such as onions, berries, kale, and green tea [60]. Flavonoids have a chemical structure of 15 carbons, with a skeleton of phenyl-benzo-γ-pyran (C6–C3–C6), also known as nucleus flava, composed of two phenyl rings and a heterocyclic (pyran) ring. Flavonoids comprise of flavones, flavonols, flavonoids, flavanones, isoflavones and anthocyanidins [61].

### Apigenin

A recent study investigated the anti-proliferative effects of apigenin, an abundant flavonoid, on the human cervical cancer cell line HeLa [62]. Initial research demonstrated an IC_50_ of 15 μM – similar to that observed for some common chemotherapeutics. Reduced viability was associated with an apoptotic profile as determined by annexin V and propidium iodide positivity. This was demonstrated by Western blot where apigenin increased the expression of Bax and decreased the expression of Bcl-2, commonly observed in apoptosis. The authors concluded that apigenin has apparent potential as a chemotherapeutic agent [62]. Erdogan et al*.* [63] observed apigenin reduce prostate cancer stem cell survival and migration, via treating PC3 and cancer stem cells with a series of μM concentrations of apigenin for 48 hours. In a dose-dependent manner, apigenin inhibited PC3 and cancer stem cell survival, that was associated with PI3K, AKT and NF-κB downregulation, and increases in p27 and p21. Apigenin significantly suppressed the migration rate of CD44+ cancer stem cells, as determined by a wound healing assay. This supressed migration was through downregulation of matrix metallopeptidases-2, -9, Snail and Slug. Thus, Erdogan et al*.* [63] highlighted apigenin to potentially prevent the proliferation and migration of cancer cells. Xu et al*.* [64] demonstrated apigenin to suppress colorectal cancer cell proliferation, migration and invasion through the inhibition of the Wnt/β-catenin signalling pathway. The occurrence of human tumorigenesis can be significantly affected by abnormal activation of the Wnt/β-catenin signalling pathway. Xu et al*.* [64] demonstrated antiproliferation effects of apigenin on HCT15 and SW480 colorectal cancer cell lines in vitro. It was determined that apigenin significantly reduced HCT15 and SW480 cell proliferation at an IC_50_ of 23.57 μM and 18.17 μM, respectively.

### Luteolin-7-O-glucoside

Maatouk et al*.* [65••] investigated the protective role of luteolin-7-O-glucoside on oxidative stress in addition to DNA damage induced by cisplatin through comet assay. Balb/c mice were injected with 10 mg/kg of cisplatin following luteolin-7-O-glucoside treatment (40 mg/kg). The results demonstrated that luteolin-7-O-glucoside attenuates the genotoxicity associated with cisplatin [65••]. This included a reduction in markers of tissue damage (creatinine, interferon γ) and oxidative stress (malondialdehyde, catalase, glutathione peroxidase, superoxide dismutase, and glutathione). Work on the liver cancer cell line HepG2 demonstrated that exposure to luteolin 7-glucoside and apigenin 7-glucoside reduced cell viability, with an IC_50_ of 21 μg/mL and 17 μg/mL, respectively. These compounds reduced the expression of NF-κB, a key pathway in the chronic inflammation associated with hepatocellular carcinoma [66].

### Hydroxytyrosol

Hydroxytyrosol was revealed to possess antibacterial, antioxidative, and anti-inflammatory properties [51]. Evidence further demonstrated effective chemotherapeutic properties through affecting several signalling pathways [67]; notably growth factor receptors [52, 68, 69], receptor support proteins [70, 71] and interleukin pathways [72]. Inhibition of cyclin D1 is core to hydroxytyrosol efficacy, resulting in cell cycle arrest at G1/S phase in the MCF-7 cell line [7]. Several studies have referenced cyclin D1 downregulation following hydroxytyrosol treatment in many cancer cell lines, including breast cancer (MCF-7, MB231) [7, 71], colon cancer (Caco-2) [73], and thyroid cancer (TPC-1, FB-2, WRO) [74].

Furthermore, hydroxytyrosol has exhibited protection to peripheral blood mononuclear cells from hydrogen peroxide-induced DNA damage and prevention of endoplasmic reticulum stress in hepatocellular carcinoma Hep G2 [75, 76]. Conversely, hydroxytyrosol has demonstrated ROS production, leading to apoptotic cell death and mitochondrial dysfunction in DLD1 colon cancer cells [77]. Moreover, evidence suggests that hydroxytyrosol can cause superoxide and hydrogen peroxide generation leading to induction of apoptosis in prostate cancer PC3 cells [78].

Zubair et al*.* [79] tested hydroxytyrosol on normal prostate epithelial cells (PWLE2 and RWPE1) alongside cancerous cells (LNCaP and C4-2), demonstrating inhibition of proliferation in a dose-dependent manner. The study revealed hydroxytyrosol inhibited cyclins D1/E and cyclin-dependent kinases cdk2/4 and induced the cell cycle inhibitors p21/p27, resulting in G1/S cell cycle arrest [79]. Hydroxytyrosol induced apoptosis, as demonstrated through caspase activation, PARP cleavage, and BAX/Bcl-2 ratio. Phosphorylation of Akt/STAT3 was inhibited and cytoplasmic retention of NF-kB was induced, which relates to the induction of apoptosis. Prostate cancers usually retain androgen receptor signalling and are normally dependent on activated Akt, NF-kB, and STAT3 signalling [80–82]. Hydroxytyrosol exhibits a pleiotropic activity against these signalling pathways leading to cell cycle arrest [79]. Terzuoli et al*.* [69] demonstrated that hydroxytyrosol significantly downregulates epidermal growth factor receptor expression in human colorectal adenocarcinoma cells (HT-29, WiDr and CaCo2). They concluded that hydroxytyrosol downregulated receptor expression through proteasomal and lysosomal degradation via receptor ubiquitination. This led Terzuoli et al*.* [69] to highlight the potential of hydroxytyrosol as a novel colon tumour treatment.

### Verbascoside

Verbascoside has demonstrated anti-tumour effects in some human cancers. Apoptosis promotion by verbascoside is associated with HIPK2, p53, HIF-1α and Rac-1 in colorectal cancer cell lines [83, 84], in addition to downregulation of STAT3, epithelial-to-mesenchymal transition (EMT) markers (vimentin, snail and zeb1) and c-Met in glioblastomas [85, 86]. Hei et al. [85] further demonstrated the downregulation of the EMT markers and c-Met in an orthotopic glioblastoma xenograft mouse model. EMT is a fundamental hallmark of metastatic tumourigenesis and has an essential role in glioma aggressiveness [87]. Hei et al*.* [85] highlighted that verbascoside can bind directly to the c-Met protein, and that verbascoside causes c-Met protein degradation through the ubiquitination-proteasome pathway. Furthermore, verbascoside was able to suppress tumour growth and enhance survival in mice [85]. This model demonstrated that verbascoside exerted effects via the same mechanisms in vivo as it did in vitro, by suppressing c-Met-mediated EMT and inducing cancer death.

## Triterpenoids

Triterpenoids are structurally varied organic compounds, made of a basic backbone modified in a multitude of ways, creating the formation of over 20,000 naturally occurring triterpenoids [88]. Triterpenoids are characterised by 30 carbon atoms, polymerised to form six isoprene units. Biosynthesis of triterpenoids occurs when its precursor squalene undergoes cyclization. Triterpenoids chemical structure is grouped from linear, through to pentacyclic [89]. Maslinic acid, oleanolic acid, erythrodiol and uvaol are the most abundant triterpenes in olive tree products [90].

### Maslinic and Oleanolic Acid

Juan et al*.* [91] observed the effect of maslinic and oleanolic acid (two triterpenoids with similar structure) on HT-29 colon cancer cells. These compounds were examined for their effect on proliferation, necrosis and apoptosis. Maslinic acid inhibited cell growth with an IC_50_ of 101.2 μM, whilst oleanolic acid demonstrated lesser antiproliferation activity with an IC_50_ of 160.6 μM. Maslinic acid increased caspase-3-like activity that was associated with increased presence of mitochondrial ROS, whereas oleanolic acid cytotoxicity was not associated with either activated caspase-3 or ROS production [91]. Detection of increased DNA fragmentation and increase in plasma membrane permeability confirmed apoptosis by maslinic acid. Kim et al*.* [92•] investigated oleanolic acid-induced cancer cell death, apoptotic mechanisms, cell cycle status, and MAPK kinase signalling in MCF-7, DU145 (prostate cancer) and U87 (human glioblastoma) cell lines. The IC_50_ values for oleanolic acid-induced cytotoxicity were 132.29 in MCF-7, 112.57 in DU145 and 163.60 in U87 cells. At 100 μg/mL oleanolic acid, there was an increased number of apoptotic cells to 27.0% in MCF-7, 27.0% in DU145 and 15.7% in U87, when compared to control cells [92•]. This greater apoptosis was a result of increased p53, cytochrome c, Bax, PARP-1 and caspase-3 expression in the cell lines. Furthermore, the different cancer lines arrested at varying phases of the cell cycle, MCF-7 and U87 cells arrested in G1, whereas DU145 cells arrested in G2 [92•]. This suggests that oleanolic acid alters the expression of the cell cycle regulatory proteins inconsistently in different types of cancer cell lines.

## Conclusion

Interest in *Olea europaea* products is increasingly researched for their beneficial effect on human health. The polyphenols detected in these products are of growing interest due to their effect on ROS production. However, this is not their only means of inhibiting cell proliferation in cancer. Natural plant polyphenols have demonstrated the ability to alter the level of ROS, either protecting biomolecules from oxidative damage (e.g., luteolin-7-O-glucoside) or inducing oxidative damage (e.g., maslinic acid). Oleuropein, hydroxytyrosol and triterpenoids are abundant in *Olea europaea* products. These polyphenolic compounds demonstrate powerful anti-oxidant, anti-angiogenic, chemotherapeutic and anti-inflammatory characteristics. Despite the continued positive results from in vitro studies on the beneficial properties of *Olive europaea* products, further in vivo investigation is needed. Initial results in animal models are promising but need to be translated to clinical setting. Treatments utilising these compounds are likely to be well tolerated, as initial animal experimentation has demonstrated [43, 50, 65, 85]. Nevertheless, initial investigation is encouraging in relation to the prevention and treatment of cancer.

## Abbreviations

GLUT: glucose transporters
IC50: half maximal inhibitory concentration
OLE: olive leaf extract
PARP: poly-ADP ribose polymerase
ROS: reactive oxygen species
